# Valorization of Citrus Peel Byproducts: A Sustainable Approach to Nutrient-Rich Jam Production

**DOI:** 10.3390/foods14081339

**Published:** 2025-04-13

**Authors:** Monica Negrea, Ileana Cocan, Calin Jianu, Ersilia Alexa, Adina Berbecea, Mariana-Atena Poiana, Marius Silivasan

**Affiliations:** 1 Faculty of Food Engineering, University of Life Sciences “King Mihai I” from Timisoara, Calea Aradului No. 119, 300645 Timisoara, Romania; monicanegrea@usvt.ro (M.N.); calinjianu@usvt.ro (C.J.); ersiliaalexa@usvt.ro (E.A.); marianapoiana@usvt.ro (M.-A.P.); 2 Faculty of Agriculture, University of Life Sciences “King Mihai I” from Timisoara, Calea Aradului No. 119, 300645 Timisoara, Romania; adina_berbecea@usvt.ro; 3 Faculty of Engineering and Applied Technologies, University of Life Sciences “King Mihai I” from Timisoara, Calea Aradului No. 119, 300645 Timisoara, Romania; mariussilivasan@usvt.ro

**Keywords:** citrus by-products, jam, nutrients, functional benefits, sensory attributes

## Abstract

The valorization of citrus peel byproducts presents a sustainable and innovative approach to reducing food waste while improving the nutritional content of fruit-based products. Citrus peels, a significant byproduct of the fruit juice industry, are abundant in bioactive compounds with recognized health benefits and functional properties, making them particularly suitable for jam production. The global citrus industry generates substantial amounts of waste, with peels accounting for approximately 50% of the total fruit mass. Conventional disposal methods often result in environmental concerns and the underutilization of valuable bioresources. This study aims to investigate the potential of incorporating citrus peel into jam formulations as a means of enhancing their nutritional and functional properties. Jams were prepared using a traditional processing technique (TP) incorporating citrus peel. The experimental jam variants included pomelo peel jam (PPJ), lime peel jam (LiPJ), lemon peel jam (LePJ), clementine peel jam (CPJ), orange peel jam (OPJ), and grapefruit peel jam (GPJ). All jam samples were subjected to comprehensive analyses, including assessments of chemical composition, total soluble solids (TSSs), titrable acidity (g/100 g acid citric), macro- and microelement contents, total phenolic content (TPC), total flavonoid content (TFC), and antioxidant activity using the FRAP assay. The study revealed high levels of biologically active compounds, such aspolyphenols, flavonoids, and vitamin C, in the jams, highlighting their antioxidant properties and potential health benefits. Among the jams, lemon peel jam (LePJ) exhibited the highest antioxidant activity and polyphenol content, making it a superior choice in terms of functional benefits. In terms of sensory analysis, orange peel jam (OPJ) was the most favored by consumers, demonstrating its high acceptability and potential for market success.

## 1. Introduction

Citrus fruits are among the most widely cultivated and consumed crops globally, with the majority of production concentrated in Brazil, India, Mexico, Spain, and the United States, which together account for over two-thirds of the global yield. These fruits and their processed products are recognized as valuable sources of essential vitamins, minerals, and dietary fiber, which play a crucial role in human nutrition and overall health [1].

The global citrus industry produces approximately 158.5 million tons of fruit annually, with peels constituting nearly 50% of this mass, resulting in substantial waste [2]. Traditionally, citrus peels are discarded or used as low-value animal feed, leading to environmental concerns and the underutilization of valuable phytochemical compounds including polyphenols such as polyphenols, flavonoids, carotenoids, and dietary fiber. Therefore, the valorization of citrus peel by-products remains a critical area of research for reducing food waste and enhancing sustainability within the food industry [3]. While traditionally discarded or used as low-value animal feed, citrus peels have well-documented applications in the extraction of pectin [4], essential oils [5], polyphenols [6], and fiber [7], which are valuable bioactive compounds. Numerous studies [3,4,5,6,7] have demonstrated the potential of these by-products for various industries, and recognizing these applications is essential for providing an accurate context.

One promising approach is the use of sugar preservation techniques to extend the shelf life and preserve the functional properties of citrus peel. This method not only offers a sustainable solution to food waste but also leads to the development of novel products with high nutritional and bioactive potential [8]. Recent research has focused on incorporating citrus peel into food products, enhancing their fiber and antioxidant content [9]. Jams, typically produced from fruit pulp, sugar, pectin, and citric acid, represent a cost-effective and easy-to-process product. Common varieties include grape, apricot, blueberry, mango, pineapple, strawberry, orange, and pomegranate. Jam production offers extended shelf life, allows for the use of out-of-season fruits, and is easy to store and transport. However, the by-products from juice production, such as peels and seeds, are often discarded [10].

Jams are preserved through a high sugar concentration (68–72%) and the natural acidity of the fruit, which inhibits microbial growth. The production process requires careful control of sugar, acidity, and pectin content, which interact during boiling to form a gel upon cooling [11]. Citrus peels, rich in bioactive compounds such as polyphenols, flavonoids, carotenoids, and essential oils, are known for their antioxidant, antimicrobial, and anti-inflammatory properties [12]. For example, pomelo peels are rich in phenolic acids, flavonoids, and tannins, which contribute to antioxidant activity [13]. Lemon peel is abundant in flavonoids such as hesperidin and eriocitrin, offering strong antioxidant properties that may help prevent oxidative-stress-related diseases. Furthermore, citrus peels provide essential oils, including limonene, as well as dietary fibers and minerals [11].

In terms of vitamin C, orange peel (136 mg/100 g) and lemon peel (129 mg/100 g) surpass other citrus peels such as grapefruit (61 mg/100 g), clementine (48 mg/100 g), and lime (29 mg/100 g) [11,14]. The crude fiber content is also higher in grapefruit peel (12.2%), orange and lemon peels (10.6%) compared to pomelo peel (9.8%), clementine peel (7.5%), and lime peel (6.4%). Additionally, citrus peels are good sources of essential minerals like potassium, calcium, and magnesium, along with trace elements such as iron, zinc, and copper, which are vital for various physiological functions [15]. Citrus peel has been used to obtain food products such as jams in other studies [16,17,18], but only orange peel jam has been evaluated exhaustively in terms of sensory, physicochemical, and nutritional characteristics [11].

This study aims to explore sustainable methods for transforming citrus peel waste into high-value, nutrient-enriched jams, which not only reduce food waste but also provide health-promoting products. Given the extensive research on citrus peel applications in jams, this study seeks to differentiate itself by analyzing improvements in processing techniques, nutritional benefits, and the overall sustainability impact of such products. By analyzing the chemical composition, macro- and microelement profile, TPC, TFC, and antioxidant activity of jams incorporating various citrus peels, this research intends to demonstrate the feasibility of producing functional foods from citrus by-products.

## 2. Materials and Methods

### 2.1. Preparation of Jam Samples

Citrus fruits, specifically pomelo, lime, lemon, clementine, orange, and grapefruit, were purchased from supermarkets in Timișoara, each in 1000 g quantities. These fruits were sourced from Spain, according to supermarket specifications. Upon acquisition, the fruits were temporarily stored at 12 °C in the storage facilities of the Principles and Methods of Preservation Laboratory within the Faculty of Food Engineering at ULST.

After sorting, the citrus fruits were washed and peeled. The peels were then immersed in cold water for 48 h, with the water replaced every 12 h. After this soaking period, the peels were drained well, and the mesocarp (the soft, spongy white tissue between the peel and pulp, also known as the epicarp) was removed. The cleaned peels were subsequently cut into thin strips (4–5 cm in length) and rolled into spirals, forming a coil-like shape. These citrus peel spirals were then strung together on a thread to form a chain. A mixture of water and sugar was added to a vessel and heated over low heat. Once the mixture reached boiling point, it was maintained at a simmer until a syrupy consistency was achieved. The vessel was then removed from the heat, and the string of citrus peel spirals was added. After 10–15 min, the mixture was returned to the heat, and the foam was removed. Fresh lemon juice was added to the mixture towards the end of the cooking process. The boiling process facilitated the balancing of the sugar content while preserving the product’s flavor, texture, and color. The jars used for packaging the product were filled manually. Each jar, with a capacity of 350 g, was pre-sterilized before use. The filled jars were stored in the laboratory storage area at 12 °C and 75% relative humidity until they were subjected to chemical, phytochemical, and sensory analyses.

Table 1 presents the composition and yield of jam from studied citrus fruits.

The percentage of waste generated after peeling each was as follows: peel constitutes 40–45% of the total fruit weight for grapefruit; pomelo, being significantly larger than other citrus fruits, has a peel that accounts for approximately 45–50% of the total fruit weight. Limes and lemons have a peel that represents about 30–35% of the fruit’s weight, and in the case of oranges and clementines, the waste percentage is around 40% of the initial fruit weight.

### 2.2. Determination of Proximate Composition and Energetic Values of Jam Samples

The moisture content was determined according to AOAC 925.10, which involves drying a known weight of the sample in an oven (Binder, Tuttlingen, Germany) at 105 °C until a constant weight is achieved. The difference in weight represents the moisture content. The ash content was determined using the AOAC 923.03 method, which involves ashing the sample at 550 °C (Nabertherm furnace, Lilienthal, Germany) for a specified period. The remaining inorganic residue is the ash content, which reflects the mineral substances present in the sample. Crude protein was determined by the Kjeldahl method (AOAC 984.13), where the nitrogen content of the sample is measured and then multiplied by a factor of 6.25 to estimate the protein content. Digestion was performed using a Velp Scientifica system (Usmate Velate, Italy), and nitrogen was quantified using a Büchi (Flawil, Switzerland) distillation unit. Crude fat was determined using the AOAC 945.16 method, in which a Soxhlet extractor (LabTech—Hamburg, Germany) was used to extract fat from the sample with hexane (Sigma-Aldrich Chemie GmbH, Munich, Germany), the fat content being quantified by solvent recovery. Total carbohydrates were calculated by difference (100% minus the sum of moisture, ash, protein, fat, and fiber content), following the AOAC 978.10 method. Reducing sugars were determined using the AOAC 967.17 method, which involves titration with Fng’s solution (Sigma-Aldrich—St. Louis, MO, USA). The energy value of the jam samples was calculated by applying Atwater factors: 4 kcal per gram of protein and carbohydrate, and 9 kcal per gram of fat (AOAC 945.30) [19].

### 2.3. Determination of Functional Properties of Jam Samples

#### 2.3.1. Preparation of the Alcoholic Extract

One gram of each jam sample (PPJ, LiPJ, LePJ, CPJ, OPJ and GPJ) was measured and placed into tubes with caps. Then, 10 mL of 70% ethanol (SC Chimreactiv SRL, Bucharest, Romania) was added. After securing the caps, the tubes were agitated for 30 min using a Holt mechanical shaker (IDL, Freising, Germany). The resulting extracts were then filtered using Whatman filter paper.

#### 2.3.2. Determination of Total Polyphenols Content (TPC)

The total polyphenol content (TPC) of six citrus peel jam extracts was assessed using the Folin–Ciocalteu method. For each extract, 0.5 mL was combined with 1.25 mL of Folin–Ciocalteu reagent (diluted 1:10 in water) (Sigma-Aldrich Chemie GmbH, Munich, Germany) and allowed to sit at room temperature for 5 min. Afterward, 1 mL of a 60 g/L Na_2_CO_3_ solution (Geyer GmbH, Renningen, Germany) was added. The samples were incubated at 50 °C for 30 min using a thermostat (INB500, Memmert GmbH, Schwabach, Germany). Absorbance was then recorded at 750 nm with a UV–VIS spectrophotometer (Specord 205; Analytik Jena AG, Jena, Germany). Each sample was tested in triplicate, and the results were presented as mg GAE/kg d.w. [20].

The calibration curve was generated using gallic acid as the standard (0–200 µg/mL), yielding the calibration equation y = 0.0174x + 0.1224 (R^2^ = 0.9986).

#### 2.3.3. Determination of Total Flavonoids Content (TFC)

The total flavonoid content was obtained following the method outlined by Dossa et al. [21], with slight changes. Briefly, 4 mL of distilled water, 0.3 mL solution of 5% NaNO_2_, and 0.3 mL solution of 10% AlCl_3_ were added to 1 mL of jam extract. After 6 min of rest, 2 mL of 1 M NaOH solution was added, the final volume being adjusted to 10 mL using 70% ethanol. The mixture was left for 15 min at room temperature before absorbance was measured at 510 nm using a UV–VIS spectrophotometer (Specord 205; Analytik Jena AG, Jena, Germany), with a 70% ethanol solution as the reference. The results, expressed as the mean in mg QUE/100 g ±SD, were based on triplicate analysis. A calibration curve was prepared with quercetin (Sigma-Aldrich Chemie GmbH, Munich, Germany) at concentrations between 5 and 100 μg/mL, with the equation y = 0.0051x + 0.6312 (R^2^ = 0.9995).

#### 2.3.4. Determination of Vitamin C Using the DCPIP Method

For vitamin C determination, the 2,6-dichloroindophenol (DCPIP) titrimetric method was used (AOAC Method 967.21). Briefly, 10 g of jam was homogenized with 50 mL of 2% hydrochloric acid solution (Sigma-Aldrich Chemie GmbH, Munich, Germany), then left to sit for 10 min; then the extract was filtered and the volume was adjusted to 100 mL with the acid solution. A measure of 10 mL of the filtered extract was pipetted into an Erlenmeyer flask and titrated with the DCPIP (Sigma-Aldrich Chemie GmbH, Munich, Germany) solution until a persistent light pink color appeared, indicating the endpoint. The concentration of vitamin C in the jam sample was determined using Equation (1) [22].

(1) $$C=\frac{V_{DCPIP}\times F\times M}{V_{sample}}$$

where

***C*** = Vitamin C concentration in the sample (mg/100 g)

***V_DCPIP_*** = Volume of DCPIP solution used for titration (mL)

***F*** = Titration factor (mg of ascorbic acid per mL of DCPIP solution, determined from standardization)

***M*** = Dilution factor (if the sample is not diluted, M = 1)

***V_sample_*** = Volume of sample solution used in titration (mL)

#### 2.3.5. Antioxidant Capacity Using Ferric Reducing Antioxidant Power (FRAP) Assay

The antioxidant activity of the samples was assessed using the FRAP assay, based on the method by Benzie and Strain [23]. A measure of 1 g of sample was mixed with 10 mL of 70% ethanol and stirred for 30 min, then filtered, and the filtrate was used for analysis. The assay measures the reduction of Fe (III) to Fe (II) in the presence of TPTZ, forming a blue Fe (II)–TPTZ complex with maximum absorbance at 593 nm [24]. Absorbance at 593 nm was recorded using a UV–VIS spectrophotometer (Specord 200 from Analytik Jena Inc. (Jena, Germany)). A 0.5 mL aliquot of diluted filtrate (1:10 with water) was mixed with FRAP reagent, and absorbance was measured after 30 min. A calibration curve was generated with FeSO_4_·7H_2_O solutions (0.05–0.5 µM Fe^2+^). Results were expressed as µM Fe^2+^ equivalents per 100 g, with triplicate analysis, and reported as the average ± SD.

#### 2.3.6. Antioxidant Capacity by 1,1-diphenyl-2-picrylhydrazyl (DPPH) Assay

To evaluate antioxidant activity (AA), a 0.03 mM DPPH ethanolic solution (Sigma-Aldrich, Germany) was used; 1 mL of extract was mixed with 2.5 mL of DPPH solution, shaken, and incubated for 30 min in the dark at room temperature. Absorbance was measured at 518 nm using a UV–VIS spectrophotometer (Specord 205, Analytik Jena, Germany). Ethanol (70%) served as a reference. The sample was tested in triplicate, and the mean value was reported. AA was calculated using Equation (2):

(2) $$\mathrm{RSA}\;(\%)\;=\;\frac{Acontrol-Asamples}{Acontrol}\cdot 100\%$$

where *A_control_* is the absorbance of the control sample and *A_sample_* is the absorbance of the sample.

The antioxidant capacity of the samples was quantified as the IC_50_ value and compared with the ascorbic acid standard [20].

### 2.4. Determination of Macro- and Microelement Profile of Citrus By-Product Jam Samples

The composition of macro- and microelements was measured after sample mineralization at 550 °C through ashing in a furnace (Nabertherm furnace, Germany) and extraction with 20% HCl (Sigma-Aldrich Chemie GmbH, Munich, Germany). The procedure followed for quantifying the major macro- and microelements was atomic absorption spectroscopy (AAS). The identification and quantification technique follows the method described by Posta et al. [25].

### 2.5. Determination of Titratable Acidity by Direct Titration (Expressed as Citric Acid—g/100 g)

The titrimetric method was employed to assess the acidic properties of citrus by-product jams, according to the method described by Kumar and Kumar [26]. Briefly, 20 g of the jam sample was homogenized in a Berzelius flask, then transferred to a 250 mL volumetric flask, and diluted with distilled water to ¾ of the flask’s capacity. The samples were heated at 80 °C for 10–15 min, cooled to room temperature, and filtered through Whatman filter paper. A 50 mL aliquot of the filtered sample was titrated with 0.1 N NaOH (Sigma-Aldrich Chemie GmbH, Munich, Germany) using phenolphthalein (Sigma-Aldrich Chemie GmbH, Munich, Germany) as the indicator. The acidity in percentage was calculated using the following formula:

(3) $$x=\frac{n\cdot K\cdot d}{a\cdot c}\cdot 100\;[\%]$$

where

*n* = volume of NaOH used (mL)

*K* = titration factor (0.0044 for citric acid)

*d* = flask volume

*a* = sample weight (g)

*c* = extract volume for titration (mL).

### 2.6. Sensory Evaluation of Jam Samples

Six jams made from citrus by-products (PPJ, LIPJ, LEPJ, CPJ, OPJ, and GPJ) were evaluated by a sensory panel consisting of 31 semi-trained evaluators (12 males and 19 females) aged between 20 and 42 years, all non-smokers and without known food allergies. The evaluations took place under standardized sensory conditions: samples were presented in identical, transparent glass cups coded with double-digit identifiers to ensure anonymity, and were served at room temperature under uniform lighting. The panel assessed sensory attributes including appearance, flavor, taste, consistency, and overall acceptability, using a 5-point hedonic scale (where 1 = “extreme dislike” and 5 = “extreme liking”). The method was adapted following the method of Emelike et al. [27].

### 2.7. Statistical Analysis

All measurements were conducted in triplicate, and the results are expressed as means ± standard deviation (SD). The differences between means were evaluated using multiple comparison tests (two-sample *t*-test assuming equal variances) in Microsoft Excel 365 (Version 2208, Redmond, WA, USA). A significance level of *p* < 0.05 was used to determine statistical significance.

## 3. Results

### 3.1. Results of Proximate Composition and Energetic Values of Jam Samples

This study aimed to valorize the by-products resulting from the fruit processing industry and to create a functional product rich in bioactive compounds.

Thus, the proximate composition (moisture, ash, total proteins, total lipids and carbohydrates), energy value and content of some individual mineral elements of the jam are presented in Table 2.

The obtained jam samples (PP, LiPJ, LePJ, CPJ, OPJ and GPJ) highlighted a moisture content ranging from 27.723 to 28.697 g/100 g, with significant differences (*p* < 0.05) between PPJ, OPJ and the rest of the jam samples analyzed (Table 2). Moisture content is a parameter indicating the shelf life of the products. A high moisture content indicates a shorter shelf life [28]. The moisture content recorded is similar to that recorded in other literature studies [1]. The highest mineral content was recorded for OPJ (4.133 g/100 g) and the lowest for GPJ (2.633 g/100 g); in this case, except for PPj, CPJ and GPJ samples, statistically significant differences were recorded. On the other hand, the jam was low in protein (0.010–1.450 g/100 g) and lipids (0.025–0.300 g/100 g). Statistically, there were significant differences in protein between all jam samples analyzed, and significant differences in lipids PPj, LiPJ and GPJ between all other samples (*p* < 0.05) (Table 2).

Higher content was recorded for carbohydrates (66.390–69.786 g/100 g) but also for sugars (65.540–67.246 g/100 g). For these characteristics, except LiPJ and LePJ, but also PPJ and GPJ, statistically significant differences (*p* < 0.05) were recorded for the other samples.

The energy value recorded for the jam samples analyzed ranged from 272,724 to 283,324 kcal/100 g (Table 2).

### 3.2. Results of Functional Properties of Studies Jam Samples

#### 3.2.1. Results of Total Polyphenols Content (TPC)

**Figure 1:**
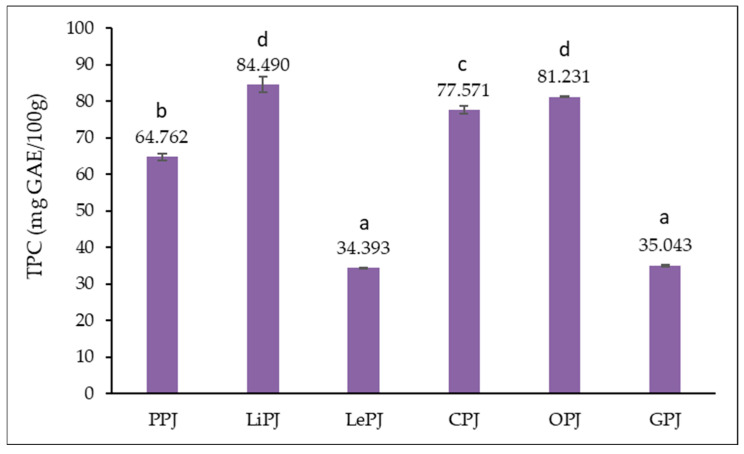
Total polyphenolic content (TPC) of jam samples. The results for the TPC are presented as the mean value of three determinations ± standard deviation (SD). According to the *t*-test, samples marked with different letters (a–d) in the same column indicate significant differences (*p* < 0.05) between the samples analyzed.

Figure 1 shows the TPC of the jam samples analyzed. Citrus peel polyphenols are recognized for their antioxidant properties and health benefits. They are involved in cell protection and may help prevent chronic diseases such as cardiovascular diseases and certain cancers [29].

From Figure 1, it can be noted that the total polyphenol content (TPC) recorded in the jam samples ranges between 34.393 and 84.490 mg GAE/100 g, the lowest value being recorded for LePJ and the highest value for LiPJ. Statistically significant differences (*p* < 0.05) are recorded between the analyzed samples except for LePJ and GPJ samples but also between LiPJ and OPJ.

#### 3.2.2. Results of Total Flavonoid Content (TFC)

**Figure 2:**
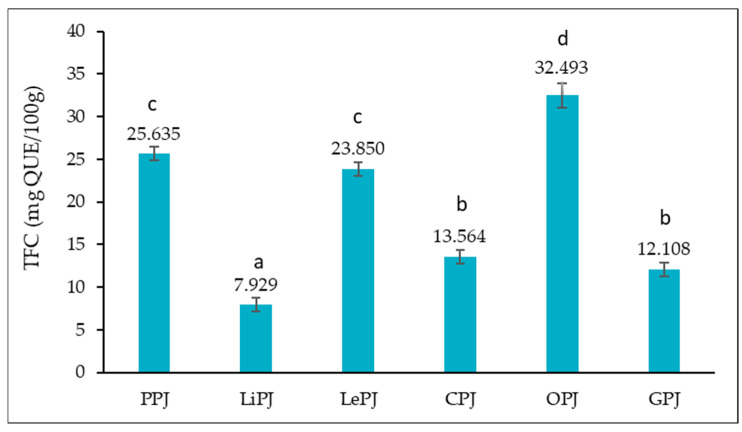
Total flavonoid content (TFC) of jam samples. The results for the TFC are presented as the mean value of three determinations ± standard deviation (SD). According to the *t*-test, samples marked with different letters (a–d) in the same column indicate significant differences (*p* < 0.05) between the samples analyzed.

In Figure 2, the total flavonoid content (TFC) of the analyzed jam samples is highlighted.

Figure 2 illustrates that the total flavonoid content (TFC) in jam samples ranged from 7.929 to 32.493 mg QUE/100 g, with the lowest value in LePJ and the highest in OPJ. Statistically significant differences (*p* < 0.05) were observed among the samples, except between PPJ and LePJ, as well as between CPJ and GPJ.

#### 3.2.3. Results of Vitamin C (mg/100 g) Content in the Studied Jam Samples

**Figure 3:**
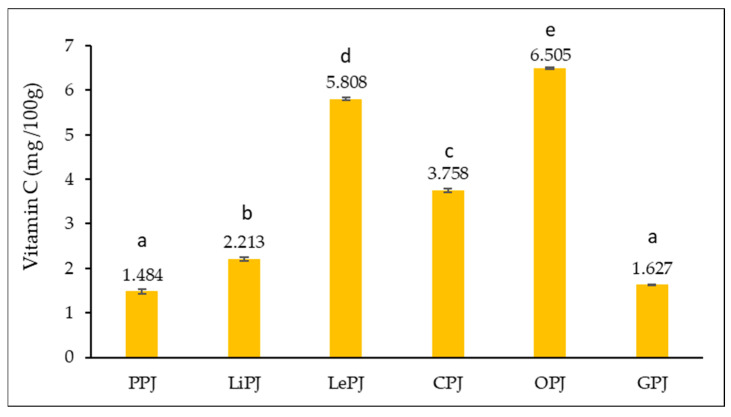
Vitamin C content of jam samples. The results for the vitamin C content are presented as the mean value of three determinations ± standard deviation (SD). According to the *t*-test, samples marked with different letters (a–e) in the same column indicate significant differences (*p* < 0.05) between the samples analyzed.

Figure 3 shows the total vitamin C content (mg/100 g) recorded in the analyzed jam samples.

From Figure 3, it can be noted that the values range from 1.484 mg/100 g (PPJ) to 6.505 mg/100 g (OPJ). Statistically, it can be observed that, except the PPJ and GPJ samples, significant differences (*p* < 0.05) were recorded between the other jam samples.

#### 3.2.4. Results of the Antioxidant Activity of the Jam Samples

The antioxidant activity of the investigated samples (Table 3) was evaluated using ferric reducing antioxidant power (FRAP) assay.

The antioxidant capacity of citrus peel jams, measured using the FRAP and DPPH assay, varied significantly among different citrus species (Table 3). Lemon peel jam (LePJ) exhibited the highest FRAP value (2319.84 ± 3.24 μM Fe^2+^/100 g), followed by orange (1464.84 ± 1.76 μM Fe^2+^/100 g) and clementine peel jam (1027.18 ± 1.92 μM Fe^2+^/100 g). In contrast, grapefruit peel jam (GPJ) had the lowest FRAP value (517.94 ± 0.74 μM Fe^2+^/100 g).

In the determination of antioxidant activity using the DPPH method, lemon peel jam (LePJ) exhibited the highest value (3.74 ± 0.13 μg/mL), followed by orange peel jam (OPJ) (3.38 ± 0.11 μg/mL) and clementine peel jam (CPL) (2.91 ± 0.09 μg/mL). In contrast, grapefruit peel jam (GPJ) showed the lowest DPPH value (2.07 ± 0.06 μg/mL).

### 3.3. Results of Macro- and Microelements Profile in Studied Jam Samples

Table 4 presents the macro- and microelement profiles of the studied citrus by-product jam.

Calcium concentrations were the highest among all analyzed minerals, ranging from 1127.94 in GPJ to 1964.69 in LiPJ, indicating its prominence in the samples. Magnesium levels were relatively high, varying from 90.57 in GPJ to 111.6 in OPJ. Potassium concentrations showed significant variation, from 126.72 in PPJ to 219.22 in OPJ. Zinc and iron also exhibited considerable differences between samples, suggesting variability in mineral composition. In contrast, copper (Cu), manganese (Mn), and sodium (Na) displayed smaller variations, indicating a more uniform distribution.

LiPJ and OPJ tended to have higher concentrations of most minerals, indicating a richer mineral composition, whereas GPJ recorded lower levels, particularly for calcium and sodium. These differences in mineral concentrations may reflect variations in sample sources, environmental conditions or manufacturing processes.

### 3.4. Results of the Acidic Properties (g/100 g Citric Acid) of Jam Samples

Table 5 presents the acidic properties of studied citrus peel jams.

Titratable acidity, expressed as citric acid (g/100 g citric acid), did not show major significant differences between the samples, with values ranging from 0.693% to 0.723%. LePJ had the lowest acidity (0.693%), while OPJ exhibited the highest value (0.723%) (Table 5). These minor variations may slightly affect the taste and stability of the jam, but the differences between samples do not seem substantial.

### 3.5. Results of the Sensory Analysis of the Jam Samples

**Figure 4:**
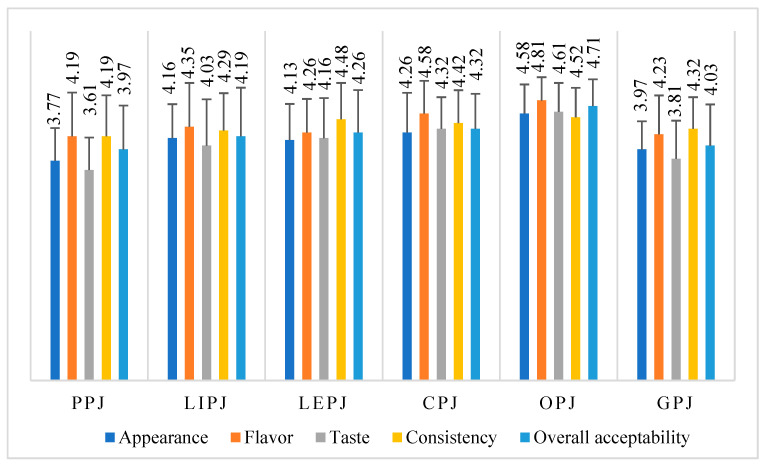
Mean values of the sensory evaluation (consumer acceptance) of citrus peel jams (PPJ—pomelo peel jam; LiPJ—lime peel jam; LePJ—lemon peel jam; CPJ—clementine peel jam; OPJ—orange peel jam; GPJ—grapefruit peel jam) using a 5-point hedonic scale (n = 31).

Consumer acceptability of citrus by-product jams (PPJ, LIPJ, LEPJ, CPJ, OPJ, and GPJ) was evaluated using sensory analysis by a panel of 31 assessors using a five-point hedonic scale. Figure 4 presents the mean scores for sensory attributes, including appearance, flavor, taste, consistency, and overall acceptability, for the six studied jam samples. The results indicate that OPJ was the most highly rated formulation, with the highest mean scores for flavor (4.81) and overall acceptability (4.71), followed by CPJ, which demonstrated balanced and consistently high ratings across all attributes, ranging from 4.26 to 4.48. LIPJ and LEPJ displayed moderate preference, with mean scores between 4.03 and 4.48. GPJ showed lower sensory acceptability, with ratings ranging from 3.81 for flavor to 4.32 for overall acceptability. Among all the formulations, PPJ was the least appreciated, receiving the lowest scores, ranging from 3.61 for flavor to 4.19 for taste and consistency.

## 4. Discussion

### 4.1. Proximate Composition and Energetic Values of Citrus By-Products Jam Samples

According to the literature, jams obtained from citrus peels are characterized by high carbohydrate and sugar contents, which is explained by the preparation technology, which involves the addition of a significant amount of sugar for preservation and textural stability. The values obtained in the present study are in agreement with those reported by Teixteira et al. [11], which indicate carbohydrate concentrations ranging from 65 to 70 g/100 g for citrus peel jams. Similarly, Lopez et al. [30] report similar values, with an average carbohydrate content of about 67.5 g/100 g for orange peel jams. These data confirm the consistency of the results obtained and their relevance in the context of the validation of the proposed recipes.

At the same time, the low protein content in the analyzed jams—a result also reflected in the literature—is a constant characteristic of this type of product. Smith et al. [31] report values between 0.3 and 1.5 g/100 g for jams obtained from citrus peels, which is in line with the data obtained in this study.

In terms of fat content, it is minimal, being between 0.05–0.10 g/100 g [31] according to the literature data, and the study by Chen et al. [32] confirms the presence of fat in concentrations below 0.1 g/100 g in citrus jams. This characteristic contributes to a relatively moderate energy value of the product, but may limit palatability and texture in some cases.

An important aspect observed in the present study is the ash content, which provides relevant information on the mineral intake of the product. The values obtained were higher than those reported in the literature, where ranges between 1.5 and 3.5 g/100 g are mentioned, according to the study by Brown et al. [33]. This suggests a more complex mineral composition and possible added functional value, especially in the context of promoting products with improved nutritional characteristics. The study by Kumar et al. [34], focusing on lemon peel jams, supports this observation, reporting an ash value of 3.1 g/100 g—at the upper limit of the literature values.

In terms of energy value, the samples analyzed fall within the range reported in previous studies, between 270 and 290 kcal/100 g. For example, Garcia et al. [35] emphasize that citrus peel jams prepared with natural sweeteners have a slightly lower energy value of about 260–275 kcal/100 g. This variation confirms that the choice of ingredients directly influences the nutritional profile of the final product. Consequently, the results obtained underline the need for a balanced formulation that responds both to sensory requirements and to current consumer trends towards low-glycemic-index and moderate-calorie products.

The data obtained are not only in line with those reported in the literature but also highlight the considerable potential applications of citrus peel jams in the development of innovative, sustainable food products adapted to modern consumer demands.

### 4.2. Functional Properties of Jam Samples

#### 4.2.1. Total Phenolic Content (TPC)

The values obtained in the present study for grapefruit (PPJ), lime (LiPJ), lemon (LePJ), clementine (CPJ), orange (OPJ) and grapefruit (GPJ) peel jams fall within the ranges previously reported in the literature, confirming the consistency and reproducibility of the experimental data. Specifically, Singh et al. [36] reported a total polyphenol content between 30 and 90 mg GAE/100 g for jams obtained from by-products resulting from citrus valorization, and the values recorded in the present study are consistent with this range.

The study conducted by Wang et al. [37] on lemon peel jams by Wang et al. [37] showed concentrations of 40–85 mg GAE/100 g, highlighting the significant impact that the processing method can have on the final content of bioactive compounds. These observations are in full agreement with our data, where, for example, lemon peel jam and clementine jam showed high concentrations of polyphenols, which can be attributed to both the characteristics of the raw material and the technological process applied.

The analysis performed by Fernandez et al. [38] on similar products reported an average content of 45–88 mg GAE/100 g, highlighting the compositional variability determined by the source of the raw material. This is particularly important as it emphasizes the role of citrus variety, ripeness and post-harvest conditions on the final phenolic profile of the product. Our study supports these conclusions, noting notable differences between jams obtained from different types of citrus peels.

Moreover, research by Turturică et al. [39], focusing on the behavior of anthocyanins during thermal processing, demonstrated that water evaporation during boiling leads to the concentration of bioactive compounds, including polyphenols, in the finished product. This observation is technologically essential, as it suggests that by optimizing the time and intensity of the heat treatment, a jam with a higher nutritional value can be obtained without compromising product stability or safety.

#### 4.2.2. Total Flavonoids Content (TFC)

Flavonoids, natural compounds present in significant amounts in citrus peels, are well known for their remarkable antioxidant properties and beneficial health effects. These bioactive substances not only provide cellular protection against oxidative stress, but are also associated with the potential to prevent chronic diseases, including cardiovascular disease and certain forms of cancer. According to the literature, some flavonoids may inhibit proteins such as RLIP76, which are involved in cancer progression, and may help reduce cholesterol levels and blood pressure, thereby alleviating cardiovascular risks. In particular, lemon peel is rich in bioactive compounds such as D-limonene, a terpene with potent antimicrobial and antifungal properties, which supports oral health and general immunity [40].

In the present study, the total flavonoid content (TFC) in jam samples ranged from 7.929 to 32.493 mg QUE/100 g, with the lowest value recorded in lemon peel jam (LePJ) and the highest in orange peel jam (OPJ). This variation reflects both the natural compositional diversity of different types of citrus peels and the influence of the processing method on the preservation of sensitive phenolic compounds.

The values obtained correlate with those reported in the literature: Chen et al. [41] reported values ranging from 5 to 35 mg QUE/100 g for citrus peel jams, while the study by Lee et al. [42] on lemon peel jams showed a range of 10–30 mg QUE/100 g, confirming the importance of the technological process in the maintenance of flavonoids. These comparisons validate the reliability of our data and confirm that the analyzed formulations fall within accepted limits while retaining significant nutritional value.

Furthermore, the variations between samples suggest clear opportunities for optimizing recipes to maximize flavonoid content in the finished product. Beyond the antioxidant value, the presence of flavonoids gives citrus peel jams excellent potential to be included in the functional diet, providing not only a pleasant taste and diversity but also real health benefits for the consumer.

#### 4.2.3. Vitamin C Content (mg/100 g)

Vitamin C (ascorbic acid) is one of the best-known natural antioxidants, essential for the proper functioning of the human body, with important roles in supporting the immune system, collagen synthesis, iron absorption and protection of cells against oxidative stress. In products obtained from citrus peels, this vitamin is present in variable amounts, significantly influenced by the type of raw material used as well as the processing method applied [43].

In the present study, the vitamin C values determined for jams obtained from various citrus peels ranged from 1.484 mg/100 g in grapefruit jam (PPJ) to 6.505 mg/100 g in orange jam (OPJ). These results are in line with those reported by Nguyen et al. [44], who indicated a range of 1–7 mg/100 g for citrus peel jams, thus reinforcing the reliability and relevance of the data obtained.

It is important to note that vitamin C is a thermolabile compound, which means that it can undergo considerable losses during the boiling and preserving processes. The study conducted by Fernandez et al. [45] draws attention to this aspect, highlighting losses of up to 40% of the initial vitamin C content during jam preparation, mainly due to high temperatures and long durations of heat treatment. These data emphasize the need for a careful technological approach when aiming at optimal preservation of sensitive bioactive compounds. In support of this idea, research published by Giannakourou et al. [46] proposes alternative processing methods, such as freeze-drying, which allow the preservation of a significantly higher proportion of vitamin C in the finished product. Such technologies, although more costly, may be viable solutions for the development of products with superior nutritional value, targeted at the market segment interested in functional foods and preventive health.

#### 4.2.4. The Antioxidant Capacity FRAP Value (μM Fe^2+^/100 g)

The determination of the antioxidant activity by FRAP (Ferric Reducing Antioxidant Power) and DPPH method provided valuable information on the reducing capacity of the compounds present in citrus peel jams, indicating their potential to neutralize free radicals and to support health by providing natural antioxidants. In the present study, the values obtained for orange peel jam (OPJ) and lemon peel jam (LePJ) samples fall within the upper range reported in the literature, demonstrating significant antioxidant activity. The antioxidant capacity of citrus peel jams, measured using the FRAP assay, varied significantly among different citrus species. Lemon peel jam (LePJ) exhibited the highest FRAP value (2319.84 ± 3.24 μM Fe^2+^/100 g), while grapefruit peel jam (GPJ) had the lowest FRAP value (517.94 ± 0.74 μM Fe^2+^/100 g). These findings align with previous studies highlighting the high flavonoid and polyphenol content in lemon and orange peels, particularly hesperidin, eriocitrin, and ascorbic acid, which contribute to their strong antioxidant activity [46,47]. Similarly, Martínez et al. [48] found that clementine peels contain high levels of phenolic acids, which enhance their reducing power. Lime (734.92 ± 1.32) and pomelo peel jams (728.86 ± 1.18) showed moderate antioxidant activity, consistent with reports that these peels contain lower flavonoid levels than lemons and oranges [49]. Processing methods may also influence antioxidant retention, as thermal treatment can degrade polyphenols [4]. The lower FRAP value of grapefruit peel jam is in line with studies showing its distinct phytochemical profile, characterized by lower flavanone and higher limonoid content, which contributes to bitterness but not necessarily a higher antioxidant capacity [15,50].

The study conducted by Peng et al. [51] on a citrus-based jelly reported a FRAP value of 28.24 μmol Fe^2+^/g, providing a point of comparison for similar products with a gelled structure. In a larger study, Wang et al. [37] determined FRAP values for orange peels ranging from 1500 to 2200 μM Fe^2+^/100 g, similar to those recorded in the present study for OPJ and LePJ samples. Also, Singh et al. [36], analyzing extracts from lemon and orange peels, reported values between 1800 and 2500 μM Fe^2+^/100 g, which are in agreement with the antioxidant activity of the LePJ sample, supporting the idea that these types of peels can be valuable sources of antioxidants in processed food products. In addition, Lee et al. [42] confirmed a wide range of 500–2300 μM Fe^2+^/100 g in a survey of several citrus peels, which highlights the variability in antioxidant content depending on variety, ripeness and raw material processing method. Determination of antioxidant activity using the DPPH (2,2-diphenyl-1-picrylhydrazyl) method has provided essential insights into the radical-scavenging capacity of citrus peel jams. This method is widely recognized in food research for the precision with which it quantifies the ability of antioxidant compounds to donate electrons, thereby neutralizing free radicals—a process essential in the prevention of cellular oxidative stress.

The IC_50_ (the concentration required to inhibit 50% of DPPH radicals) or EC_50_ (the equivalent for solid extracts) values obtained in the present study fall within the ranges reported in the literature, confirming the high bioactive potential of citrus peels used in jam recipes. For example, Bengag and Allem [52] reported an extremely low IC_50_ of 1.91 μg/mL for Citrus reticulata peel jam, suggesting a remarkable antioxidant capacity of this type of citrus, especially in extracts derived from peel. Also, Oboh and Ademosun [53] investigated the antioxidant activity of phenolic extracts from orange, grapefruit and shaddock peels, obtaining EC_50_ values of 1.7 mg/mL for orange and 1.4 mg/mL for grapefruit, denoting increased efficiency in scavenging free radicals, comparable to some of the samples in our study.

A particularly relevant result is that reported by Hussein et al. [54], who analyzed tangerine-peel-based jams and obtained an IC_50_ of 3.85 μg/mL, a value close to those determined in the present research, which confirms that conventional processing methods can largely preserve the bioactive properties of plant-derived ingredients. By analyzing the values reported in the literature and the values obtained in the present study, we can conclude that the heat treatment applied in the process of obtaining jams did not negatively influence the antioxidant activity of the obtained samples.

### 4.3. Macro- and Microelement Profiles of Jam Samples Made from Citrus By-Products

The macro- and micro-nutrient profiles determined in citrus peel jam samples reflects an important nutritional diversity, with significant implications for the formulation of functional products and the promotion of a balanced diet. The analysis of the mineral content shows a complex composition with variations depending on the type of citrus peel used, the technological process applied and the origin of the raw material.

The values obtained in this study are generally comparable with those reported in the literature, but also show differences. For example, in terms of copper (Cu) content, Amorim et al. [55] reported values between 0.20 and 0.85 ppm in grape peel jams, and Alshallash et al. [10] identified similar ranges for citrus by-products, respectively, between 0.40 and 1.00 ppm. The values obtained in this study fall within these ranges, suggesting a moderate but constant contribution of copper in these products.

For zinc (Zn), an essential element in protein synthesis and in the functioning of the immune system, the literature indicates values between 3.5 and 7.2 ppm for fruit jams (Alshallash et al. [10]) and between 2.1 and 5.8 ppm for grape skin jams (Amorim et al. [55]). The values in this research are similar, highlighting the ability of these products to provide a useful intake of zinc, particularly in dietary contexts where this micronutrient is deficient.

Iron (Fe), an essential mineral in oxygen transport and in the prevention of anemia, has been reported in the literature in the range of 5.2–10.5 ppm for plant by-product jams. The data obtained confirm the presence of this element in a nutritionally valuable range, supporting the idea that such products can be included in diets with high iron requirements.

The manganese (Mn) content in the literature is reported to be between 0.50 and 1.60 ppm in fruit peel jams [10] and between 0.75 and 1.45 ppm in grape peel jams [51]. The presence of manganese in the analyzed samples provides an additional contribution to the antioxidant activity and enzymatic functioning of the organism.

From a macroelement perspective, the content of calcium (Ca), essential for bone system health, has been reported to be between 900 and 1800 ppm in various fruit jams, and for citrus by-products, values range between 1100 and 1950 ppm [10]. Also, the magnesium (Mg) content in citrus peel jams is estimated to be between 85 and 110 ppm, while for sodium (Na), the reported values are between 30 and 55 ppm, and for potassium (K), between 140 and 210 ppm [10].

Also, the magnesium (Mg) content in citrus peel jams is estimated to be between 85 and 110 ppm, while for sodium (Na), the reported values are between 30 and 55 ppm, and for potassium (K), between 140 and 210 ppm [10].

Based on the results obtained, it can be stated that the most mineral-valuable samples are LiPJ (lime peel jam) and OPJ (orange peel jam), which showed higher concentrations of calcium, magnesium and potassium. These values highlight the potential of these varieties to be developed as functional products to support the body’s electrolyte and mineral balance. In contrast, GPJ (grapefruit peel jam) showed lower values for calcium and sodium, underlining a significant variability depending on the type of raw material.

### 4.4. Acidic Properties of Jam Samples Made from Citrus By-Products

The determination of acidic properties, in particular, the citric acid content, expressed in g/100 g, is an important indicator in assessing the sensory profile, microbiological stability and shelf life of citrus peel jams. The values obtained in this study show moderate variability between samples but are sufficiently relevant to subtly influence both taste and functional properties of the finished product.

Thus, the lemon peel jam (LePJ) sample had the lowest citric acid content of 0.693%, while the highest value was observed for orange peel jam (OPJ) at 0.723% (Table 5). Although these variations may seem minor at first glance, they have the potential to influence the flavor balance of the product as well as its long-term stability, especially in the absence of added preservatives. In addition to taste, higher acidity may also inhibit the growth of micro-organisms, thus having a dual role—sensory and technological.

These results are in agreement with the literature data. For example, Rini et al. [56] reported citric acid values ranging from 0.33% to 1.02% for various types of jams, confirming that the range identified in the present study is within the accepted limits for similar products. Moreover, research by Czech et al. [12] on citrus-based products identified a very close range between 0.68 and 0.73 g/100 g, which supports the robustness of the values obtained in the present context.

### 4.5. Sensorial Evaluation of Jam Samples Made from Citrus By-Products

The sensory evaluation of jams made from citrus by-products revealed significant differences between samples, providing essential information on consumer preferences and possible directions for optimizing recipes and technological processes. The panel of evaluators ranked the jam samples according to sensory attributes as follows: OPJ > CPJ > LiPJ ≈ LePJ > GPJ > PPJ, which clearly shows that certain types of citrus peels have superior potential in developing products with a pleasant and balanced flavor impact.

The most highly rated sample evaluated was the orange peel jam (OPJ), which obtained high average scores for all the analyzed criteria: 4.81 for aroma, 4.71 for overall acceptability, 4.61 for taste, 4.58 for consistency and 4.38 for visual appearance. These results highlight the excellent potential of orange peel in the formulation of products with high sensory qualities, being an optimal choice for the development of functional jams to meet market demands.

The clementine peel jam (CPJ) ranked second with very similar scores: 4.48 for taste, 4.42 for aroma, 4.32 for overall acceptability, 4.28 for appearance and 4.26 for consistency. This sample demonstrated an outstanding taste and visual balance, which supports its use as an alternative or complement to OPJ in products for the premium or gourmet segments.

The LiPJ (lime) and LePJ (lemon) samples achieved moderate scores, ranging from 4.03 to 4.48, suggesting an acceptable sensory preference, but one that can be improved by recipe adjustments such as balancing acidity or optimizing texture to amplify positive attributes perceived by consumers.

On the other hand, grapefruit peel jam (GPJ) was rated lower, with scores of 3.81 for flavor, 3.97 for appearance, 4.03 for overall acceptability and 4.32 for taste. These results indicate a less favorable perception, possibly related to the bitter notes characteristic of grapefruit, suggesting the need for adjustments in the heat treatment process or combination with other ingredients to mask astringency.

The lowest rated product was the grapefruit peel jam (PPJ), which obtained low scores of 3.61 for flavor, 3.77 for appearance and 4.19 for taste, consistency and overall acceptability. This result signals clear opportunities for improvement, both in terms of flavor profile and texture, perhaps by pairing with more pleasant flavors or by adjusting the degree of grinding and gelatinization.

These results are also supported by the literature. For example, Teixeira et al. (2020) [11] demonstrated that products derived from orange peel receive the highest sensory scores due to rich flavor and balanced texture. Similarly, the study by Kumar et al. (2021) [26] showed that jams formulated with citrus by-products provide an attractive sensory profile with emphasis on flavor and consistency attributes.

These findings support the conclusion that OPJ is the most sensory-appealing formulation, consistent with previous studies, while PPJ’s lower acceptability highlights areas for potential improvement in sensory optimization.

## 5. Conclusions

This study highlights the potential of citrus peel by-products in producing nutrient-rich jams with functional health benefits. The jams made from pomelo, lime, lemon, clementine, orange, and grapefruit peels demonstrated significant nutritional and bioactive properties, such as high levels of polyphenols, flavonoids, and vitamin C, offering notable antioxidant activity. Lemon peel jam showed the highest antioxidant content, while orange peel jam was most preferred in sensory tests, indicating good market potential. The jams also exhibited varying levels of essential minerals like calcium, magnesium, and potassium.

The original contribution of this study lies in its comprehensive examination of the chemical composition of citrus peels, the development of a method for producing jams from these by-products, and its assessment of the environmental and sustainability impact. By focusing on the potential of citrus peels as a sustainable food ingredient, this research contributes to a deeper understanding of their nutritional and bioactive value. Furthermore, it proposes a practical method for utilizing what would otherwise be waste, thus supporting the reduction in food waste and advancing sustainable practices in the food industry. The importance of these data demonstrates that food products made from citrus peels—often considered just agri-food waste—may have real functional value, providing consumers with natural antioxidants with potential health benefits.

## Figures and Tables

**Table 1:** Composition and yield of jam from different citrus fruits.

Fruit	Quantity (g)	Peel with Mesocarp (g)	Epicarp (g)	Sugar (g)	Water (mL)	Lemon Juice (mL)	Final Yield of Jam (g)
Pomelo	1000	450	180	270	300	30	351
Lime	1000	350	180	270	300	30	350
Lemon	1000	400	180	270	300	30	350
Clementine	1000	400	180	270	300	30	351
Orange	1000	400	180	270	300	30	351
Grapefruit	1000	425	180	270	300	30	350

**Table 2:** Proximate composition of studied jam samples and the energetic values.

Samples	Moisture Content g/100 g	Ash Content (g/100 g)	Protein (g/100 g)	Fat (g/100 g)	Carbohydrates (g/100 g)	Sugar (g/100 g)	Energy Value (kcal/100 g)
**PPJ**	27.723 ± 0.547 ^a^	2.717 ± 0.091 ^a,b^	0.650 ± 0.021 ^c^	0.070 ± 0.002 ^a^	68.840 ± 2.351 ^c^	66.458 ± 1.965 ^c^	278.59
**LiPJ**	28.127 ± 0.676 ^b^	3.767 ± 0.127 ^d^	0.400 ± 0.012 ^b^	0.060 ± 0.002 ^a^	67.646 ± 2.220 ^b^	66.025 ± 1.902 ^b^	272.724
**LePJ**	28.217 ± 0.694 ^b^	3.187 ± 0.108 ^c^	1.200 ± 0.041 ^e^	0.300 ± 0.010 ^c^	67.096 ± 2.183 ^b^	65.896 ± 1.857 ^b^	275.884
**CPJ**	28.347 ± 0.708 ^b^	2.787 ± 0.087 ^b^	0.820 ± 0.022 ^d^	0.100 ± 0.005 ^b^	69.786 ± 2.455 ^d^	67.246 ± 2.013 ^d^	283.324
**OPJ**	27.737 ± 0.151 ^a^	4.133 ± 0.132 ^e^	1.450 ± 0.046 ^f^	0.290 ± 0.010 ^c^	66.390 ± 1.887 ^a^	65.540 ± 1.754 ^a^	273.970
**GPJ**	28.697 ± 0.671 ^b^	2.633 ± 0.072 ^a^	0.010 ± 0.001 ^a^	0.025 ± 0.001 ^a^	68.635 ± 2.425 ^c^	66.320 ± 1.755 ^c^	274.805

The results for the chemical composition are presented as the mean value of three determinations ± standard deviation (SD). According to the *t*-test, samples marked with different letters (a–f) in the same column indicate significant differences (*p* < 0.05) between the samples analyzed.

**Table 3:** The antioxidant activity of samples by ferric reducing antioxidant power (FRAP) assay.

Sample	FRAP Value (μM Fe^2+^/100 g)	DPPH (IC_50_ μg/mL)
Orange peel jam (OPJ)	1464.84 ± 1.76 ^d^	3.38. ± 0.11 ^d^
Lime peel jam (LiPJ)	734.92 ± 1.32 ^b^	2.64 ± 0.08 ^b^
Lemon peel jam (LePJ)	2319.84 ± 3.24 ^e^	3.74 ± 0.13 ^e^
Pomelo peel jam (PPJ)	728.86 ± 1.18 ^b^	2.57 ± 0.07 ^b^
Clementine peel jam (CPJ)	1027.18 ±1.92 ^c^	2.91 ± 0.09 ^c^
Grapefruit peel jam (GPJ)	517.94 ±0.74 ^a^	2.07 ± 0.06 ^a^

The results are expressed as the average value of three independent analyses ± SD. According to the *t*-test, samples marked with different letters (a–e) in the same column indicate significant differences (*p* < 0.05) between the samples analyzed.

**Table 4:** Macro- and microelement profiles of the studied citrus by-product jam.

Sample	Cu	Zn	Fe	Mn	Ca	Mg	Na	K
	ppm							
PPJ	0.822 ± 0.025 ^d^	2.260 ± 0.065 ^a^	4.826 ± 0.144 ^a^	0.466 ± 0.012 ^a^	1578.780 ± 47.254 ^b^	91.600 ± 2.725 ^b^	43.03 ± 1.290 ^b^	126.720 ± 126.287 ^a^
LiPJ	0.506 ± 0.013 ^b^	5.253 ± 0.159 ^c^	13.076 ± 0.387 ^e^	1.856 ± 0.054 ^e^	1964.690 ± 57.456 ^e^	90.850 ± 2.719 ^a^	54.59 ± 1.642 ^d,e^	187.690 ± 5.578 ^d^
LePJ	0.693 ± 0.019 ^c^	5.755 ± 0.171 ^c^	5.640 ± 0.165 ^b^	0.579 ± 0.018 ^b^	1737.650 ± 51.129 ^c^	93.950 ± 2.889 ^c^	49.73 ± 1.475 ^c^	148.791 ± 4.426 ^b^
CPJ	0.950 ± 0.031 ^e^	4.039 ± 0.122 ^b^	8.590 ± 0.255 ^d^	0.804 ± 0.025 ^d^	1588.930 ± 47.875 ^b^	100.430 ± 3.015 ^d^	51.07 ± 1.528 ^c,d^	190.443 ± 5.687 ^d^
OPJ	0.308 ± 0.008 ^a^	6.519 ± 0.189 ^d^	4.959 ± 0.142 ^a^	0.779 ± 0.022 ^c^	1897.010 ± 55.910 ^d^	111.600 ± 3.351 ^e^	56.17 ± 1.672 ^e^	219.222 ± 6.527 ^e^
GPJ	0.304 ± 0.007 ^a^	6.033 ± 0.182 ^d^	6.637 ± 0.191 ^c^	0.879 ± 0.026 ^d^	1127.940 ± 34.038 ^a^	90.570 ± 2.618 ^a^	35.89 ± 1.045 ^a^	162.141 ± 4.816 ^c^

The results for the mineral composition are presented as the mean value of three determinations ± standard deviation (SD). According to the *t*-test, samples marked with different letters (a–e) in the same column indicate significant differences (*p* < 0.05) between the samples analyzed.

**Table 5:** Acidic properties (g/100 g citric acid) of citrus peel jams.

Samples	Titratable Acidity (g/100 g Citric Acid)
PPJ	0.707 ± 0.006 ^a^
LiPJ	0.700 ± 0.001 ^a^
LePJ	0.693 ± 0.006 ^a^
CPJ	0.720 ± 0.010 ^b^
OPJ	0.723 ± 0.006 ^b^
GPJ	0.707 ± 0.015 ^a^

The results for the chemical composition are presented as the mean value of three determinations ± standard deviation (SD). According to the *t*-test, samples marked with different letters (a,b) in the same column indicate significant differences (*p* < 0.05) between the samples analyzed.

## Data Availability

The original data presented in the study are openly available at the University of Life Sciences “King Mihai I” from Timisoara.

## References

[1] Casas Cardoso L., Cejudo Bastante C., Mantell Serrano C., Martínez de la Ossa E.J. (2022). Application of Citrus By-Products in the Production of Active Food Packaging. Antioxidants.

[2] World Citrus Organisation. https://worldcitrusorganisation.org/.

[3] Chavan P., Singh A.K., Kaur G. (2018). Recent progress in the utilization of industrial waste and by-products of citrus fruits: A review. J. Food Process Eng..

[4] May C.D. (1990). Industrial pectins: Sources, production and applications. Carbohydr. Polym..

[5] del Valle J.M., Calderón D., Núñez G.A. (2019). Pressure drop may negatively impact supercritical CO_2_ extraction of citrus peel essential oils in an industrial-size extraction vessel. J. Supercrit. Fluids.

[6] Fiorentini C., Duserm Garrido G., Bassani A., Cortimiglia C., Zaccone M., Montalbano L., Spigno G. (2021). Citrus peel extracts for industrial-scale production of bio-based active food packaging. Foods.

[7] Wang L., Xu H., Yuan F., Pan Q., Fan R., Gao Y. (2015). Physicochemical characterization of five types of citrus dietary fibers. Biocatal. Agric. Biotechnol..

[8] Chen Y., Pan H., Hao S., Pan D., Wang G., Yu W. (2021). Evaluation of phenolic composition and antioxidant properties of different varieties of Chinese citrus. Food Chem..

[9] Sharma P., Vishvakarma R., Gautam K., Vimal A., Gaur V.K., Farooqui A., Varjani S., Younis K. (2022). Valorization of Citrus Peel Waste for the Sustainable Production of Value-Added Products. Bioresour. Technol..

[10] Alshallash K.S., Shahat M., Ibrahim M.I., Hegazy A.I., Hamdy A.E., Elnaggar I.A., Abd El-wahed A.E.-W.N., Taha I.M. (2023). The Effect of Different Processing Methods on the Behavior of Minerals Content in Food Products. J. Ecol. Eng..

[11] Teixeira F., Santos B.A.d., Nunes G., Soares J.M., Amaral L.A.d., Souza G.H.O.d., Resende J.T.V.d., Menegassi B., Rafacho B.P.M., Schwarz K. (2020). Addition of Orange Peel in Orange Jam: Evaluation of Sensory, Physicochemical, and Nutritional Characteristics. Molecules.

[12] Czech A., Malik A., Sosnowska B., Domaradzki P. (2021). Bioactive Substances, Heavy Metals, and Antioxidant Activity in Whole Fruit, Peel, and Pulp of Citrus Fruits. Int. J. Food Sci..

[13] Saini R.K., Ranjit A., Sharma K., Prasad P., Shang X., Gowda K.G.M., Keum Y.-S. (2022). Bioactive Compounds of Citrus Fruits: A Review of Composition and Health Benefits of Carotenoids, Flavonoids, Limonoids, and Terpenes. Antioxidants.

[14] Sharma S., Singh B., Kaur G., Srivastava Y., Sandhu R.S. (2024). Nutritional, Bioactive, and Health Potential of Pomelo (*Citrus maxima*): An Exotic Underutilized Fruit. Nutr. Food Sci..

[15] Sir Elkhatim K.A., Elagib R.A., Hassan A.B. (2018). Content of phenolic compounds and vitamin C and antioxidant activity in wasted parts of Sudanese citrus fruits. Food Sci. Nutr..

[16] Garcia-Viguera C., Tomás-Barberán F.A., Ferreres F., Artés F., Tomás-Lorente F. (1993). Determination of citrus jams genuineness by flavonoid analysis. Z. Für Leb. Und-Forsch. A.

[17] Avula B., Upparapalli S.K., A Khan I. (2007). Simultaneous Analysis of Adrenergic Amines and Flavonoids in Citrus Peel Jams and Fruit Juices by Liquid Chromatography: Part 2. J. AOAC Int..

[18] Suri S., Singh A., Nema P.K., Singh Purewal S., Punia Bangar S., Kaur P. (2023). Bioactive Compounds in Citrus Fruits: Extraction and Identification. Recent Advances in Citrus Fruits.

[19] AOAC (2020). Official Methods of Analysis.

[20] Cadariu A.I., Cocan I., Negrea M., Alexa E., Obistioiu D., Hotea I., Radulov I., Poiana M.-A. (2022). Exploring the Potential of Tomato Processing Byproduct as a Natural Antioxidant in Reformulated Nitrite-Free Sausages. Sustainability.

[21] Dossa S., Negrea M., Cocan I., Berbecea A., Obistioiu D., Dragomir C., Alexa E., Rivis A. (2023). Nutritional, Physico-Chemical, Phytochemical, and Rheological Characteristics of Composite Flour Substituted by Baobab Pulp Flour (*Adansonia digitata* L.) for Bread Making. Foods.

[22] AOAC International (2016). Official Methods of Analysis.

[23] Benzie I.F.F., Strain J.J. (1996). The Ferric Reducing Ability of Plasma (FRAP) as a Measure of “Antioxidant Power”: The FRAP Assay. Anal. Biochem..

[24] Metzner Ungureanu C.-R., Poiana M.-A., Cocan I., Lupitu A.I., Alexa E., Moigradean D. (2020). Strategies to Improve the Thermo-Oxidative Stability of Sunflower Oil by Exploiting the Antioxidant Potential of Blueberries Processing Byproducts. Molecules.

[25] Poșta D.S., Radulov I., Cocan I., Berbecea A.A., Alexa E., Hotea I., Iordănescu O.A., Băla M., Cântar I.C., Rózsa S. (2022). Hazelnuts (*Corylus avellana* L.) from Spontaneous Flora of the West Part of Romania: A Source of Nutrients for Locals. Agronomy.

[26] Kumar P., Kumar M. (2017). Quality assessment and determination of acidity in fruit jams. Int. J. Food Sci. Technol..

[27] Emelike N.J.T., Akusu O.M. (2019). Quality Attributes of Jams and Marmalades Produced from Some Selected Tropical Fruits. J. Food Process Technol..

[28] Medina M.S., Tudela J.A., Marín A., Allende A., Gil M.I. (2012). Short postharvest storage under low relative humidity improves quality and shelf life of minimally processed baby spinach (*Spinacia oleracea* L.). Postharvest Biol. Technol..

[29] Zhao X.J., Xing T.T., Li Y.F., Jiao B.N. (2019). Analysis of Phytochemical Contributors to Antioxidant Capacity of the Peel of Chinese Mandarin and Orange Varieties. Int. J. Food Sci. Nutr..

[30] Lopez D., Ramirez C., Ortega S. (2022). Carbohydrate content in citrus peel jams: A comparative study. Food Sci. Res. J..

[31] Smith B., Collins R., Murphy T. (2020). Protein and mineral content in fruit preserves: A systematic review. Nutr. Food Sci..

[32] Chen J., Huang L., Zhou M. (2021). Lipid content and caloric value of citrus-based jams. J. Food Biochem..

[33] Brown H., Jackson P., Williams T. (2020). Ash content and mineral analysis of citrus-based food products. J. Food Compos. Anal..

[34] Kumar N., Sharma D., Verma S. (2021). Mineral composition and bioavailability in lemon peel jams. Food Chem. Adv..

[35] Garcia M., Torres J., Hernandez F. (2023). Energy value and sugar composition of citrus-based sweet spreads. J. Funct. Foods.

[36] Singh A., Patel R., Sharma P. (2022). Flavonoid content and antioxidant properties of citrus fruit-based products. J. Agric. Food Chem..

[37] Wang Y., Chen X., Li Z. (2023). Polyphenolic composition and antioxidant activity of citrus peels and jams: A comparative analysis. Food Chem..

[38] Fernandez R., Lopez M., Gomez A. (2022). Effect of thermal processing on ascorbic acid retention in citrus preserves. Int. J. Food Preserv..

[39] Turturică M., Stănciuc N., Mureșan C., Râpeanu G., Croitoru C. (2018). Thermal Degradation of Plum Anthocyanins: Comparison of Kinetics from Simple to Natural Systems. J. Food Qual..

[40] Young J.F., Dragstedt L.O., Haraldsdóttir J., Daneshvar B., Kall M.A., Loft S., Nilsson L., Nielsen S.E., Mayer B., Skibsted L.H. (2002). Green Tea Extract Only Affects Markers of Oxidative Status Postprandially: Lasting Antioxidant Effect of Flavonoid-Free Diet. Br. J. Nutr..

[41] Chen H., Zeng J., Wang B., Cheng Z., Xu J., Gao W., Chen K. (2021). Structural Characterization and Antioxidant Activities of *Bletilla striata* Polysaccharide Extracted by Different Methods. Carbohydr. Polym..

[42] Lee K., Park S., Choi Y. (2022). Flavonoid stability in citrus peel-derived jams during storage. Int. J. Food Sci. Technol..

[43] Carr A.C., Maggini S. (2017). Vitamin C and immune function. Nutrients.

[44] Nguyen T., Le H., Tran P. (2023). Vitamin C stability in citrus-based jams under different processing conditions. J. Food Sci. Technol..

[45] Giannakourou M.C., Taoukis P.S. (2021). Effect of Alternative Preservation Steps and Storage on Vitamin C Stability in Fruit and Vegetable Products: Critical Review and Kinetic Modelling Approaches. Foods.

[46] Sharma K., Mahato N., Lee Y.R., Cho M.H. (2020). Hesperidin and other bioactive flavonoids from lemon peel: Antioxidant and health benefits. Phytochem. Rev..

[47] Rafique N., Mamoona T., Ashraf N., Hussain S., Ahmed F., Ali Shah T., Bourhia M. (2023). Exploring the Nutritional and Sensory Potential of Karonda Fruit: Physicochemical Properties, Jam Production, and Quality Evaluation. Food Sci. Nutr..

[48] Martinez L., Delgado C., Ruiz M. (2021). Soluble solids and acidity in citrus preserves: Quality indicators. Food Qual. Prefer..

[49] Huang Y., Wang X., Liu Q. (2017). Flavonoid composition and antioxidant properties of citrus peels. J. Agric. Food Chem..

[50] Singh B., Singh J.P., Kaur A., Singh N. (2020). Phenolic composition, antioxidant potential and health benefits of citrus peel. Food Res. Int..

[51] Peng M., Gao Z., Liao Y., Guo J., Shan Y. (2022). Development of citrus-based functional jelly and an investigation of its anti-obesity and antioxidant properties. Antioxidants.

[52] Bengag A., Allem R. (2016). Evaluation of antioxidant potential of citrus peel essence from chlef region (algeria). Int. J. Pharma Bio Sci..

[53] Oboh G., Ademosun A.O. (2012). Characterization of the antioxidant properties of phenolic extracts from some citrus peels. J. Food Sci. Technol..

[54] Hussein A.M., Kamil M.M., Hegazy N.A., Mahmoud K.F., Ibrahim M.A. (2015). Utilization of some fruits and vegetables by-products to produce high dietary fiber jam. Food Sci. Qual. Manag..

[55] Amorim K.D., Alves G.H., de Carvalho M.V., Pires L.L., Perrone D. (2019). Grape Peel (Syrah Var.) Jam as a Polyphenol-Enriched Functional Food Ingredient. Food Sci. Nutr..

[56] Rini S., Gindi A., Chua S., Lun P., Koh C.-C., Hii S.L. (2019). Optimizing the Acceptability of Jam from Baccaurea Angulata Fruit Peel. J. Soc. Sci. Humanit..

